# On the Current Conduction and Interface Passivation of Graphene–Insulator–Silicon Solar Cells

**DOI:** 10.3390/nano15060416

**Published:** 2025-03-08

**Authors:** Hei Wong, Jieqiong Zhang, Jun Liu, Muhammad Abid Anwar

**Affiliations:** 1 Department of Electrical Engineering, City University of Hong Kong, Hong Kong, China; 2 Hubei Jiu Feng Shan Laboratory, Wuhan 430074, China; zhangjieqiong@jfslab.com.cn (J.Z.); liujun@jfslab.com.cn (J.L.); 3 School of Information Sciences and Electronic Engineering, Zhejiang University, Hangzhou 310027, China; abid_anwar@zju.edu.cn

**Keywords:** Schottky junction, solar cell, metal–insulator–semiconductor, direct tunneling, Fowler–Nordheim tunneling, Si dangling bond, high-k oxide

## Abstract

Interface-passivated graphene/silicon Schottky junction solar cells have demonstrated promising features with improved stability and power conversion efficiency (PCE). However, there are some misunderstandings in the literature regarding some of the working mechanisms and the impacts of the silicon/insulator interface. Specifically, attributing performance improvement to oxygen vacancies and characterizing performance using Schottky barrier height and ideality factor might not be the most accurate or appropriate. This work uses Al_2_O_3_ as an example to provide a detailed discussion on the interface ALD growth of Al_2_O_3_ on silicon and its impact on graphene electrode metal–insulator–semiconductor (MIS) solar cells. We further suggest that the current conduction in MIS solar cells with an insulating layer of 2 to 3 nm thickness is better described by direct tunneling, Poole–Frenkel emission, and Fowler–Nordheim tunneling, as the junction voltage sweeps from negative to a larger forward bias. The dielectric film thickness, its band offset with Si, and the interface roughness, are key factors to consider for process optimization.

## 1. Introduction

Graphene/Si solar cells have shown promising features for photovoltaic applications because of the high transmittance, high carrier mobility, and flexibility of this 2D material [1,2,3,4,5,6,7]. However, the graphene/Si interface is chemically and electronically unstable, which gives rise to long-term stability and limits the power conversion efficiency of solar cells [5,6,7,8]. The first Gr/Si solar cell demonstrated by Li et al. has an efficiency of 1.5% only [9]. Several tactics for characteristic enhancements have been proposed [5,6,7,8,10,11,12,13,14,15,16]. Several reports confirmed that an insulating interlayer between the silicon and graphene film or a metal–insulator–semiconductor (MIS) structure enhances the stability and performance of graphene/Si Schottky junctions [8,10,11,12,13,14,15,16]. Kadam et al. found that the efficiency of the solar cell improved by 12.31% by inserting a 2 nm thick HfO_2_ in between the graphene and silicon [13]. With the additional top layer passivation, the efficiency can be further enhanced up to 14.01%.

Among the possible passivation materials, aluminum oxide (Al_2_O_3_) is considered one of the most promising candidates [14,15,16]. Aluminum oxide has high thermal stability in terms of the thin film itself and its interface to the silicon, good adhesion to two-dimensional materials, and can be deposited by atomic layer deposition (ALD) with precise control over the film thickness and uniformity [16,17,18,19]. Kim and co-workers conducted a comparative study for Al_2_O_3_ growth with NH_3_, H_2_O_2_, and NH_3_–H_2_O_2_ dual precursors [16]. They found that the power conversion efficiency (PCE) of the Gr/Al_2_O_3_/Si structure can be enhanced up to 9.49% if the Al_2_O_3_ film is a deposited NH_3_ -H_2_O_2_ dual precursor compared to the 3.19% PCE for the Gr/Si solar cell without any interfacial layer [16]. Kim et al. attributed the improvement to the oxygen vacancy reduction based on the observation of a reduced 532.5 eV O 1s X-ray photoelectron spectroscopy (XPS) signal, and the current conduction of the MIS structure was attributed to the Schottky current. These explanations are incorrect. Indeed, recent reports on Gr/insulator/Si solar cells have developed several misconceptions, leading to misunderstandings about their operational mechanisms and optimization processes. Firstly, the 532.5 eV O 1s peak is not indicative of oxygen vacancies. Secondly, current conduction in the Gr/insulator/Si MIS structure is better described by tunneling mechanisms rather than Schottky emission. Thirdly, besides oxygen vacancies, the thin Al_2_O_3_ layer (~1 nm) can induce an additional field effect due to the fixed negative charge in the Al_2_O_3_ film [20]. Fourthly, the atomic layer deposition (ALD) of Al_2_O_3_ on silicon typically begins with hydrogen-terminated Si substrates, resulting in an interfacial transition layer [17,18,19]. The different characteristics arising from various ALD recipes or precursors are more related to the Al_2_O_3_/Si interface than to the bulk properties of the Al_2_O_3_ film alone.

This work aims to provide a comprehensive study, incorporating additional experimental findings and deeper insights into the silicon/Al_2_O_3_ interface physics, focusing on the characteristics of Gr/Al_2_O_3_/Si solar cells. In Section 2, we will discuss the electronic nature of the Gr/Si interface, exploring the photosensitivity of the Gr/Si Schottky diode. Section 3 will cover the physicochemical nature of silicon surface passivation with an ALD Al_2_O_3_ layer, including the interface growth of ALD-deposited Al_2_O_3_ on HF-treated silicon surfaces and the nature of the O 1s XPS peak in the energy range of 532 to 534 eV. The current conduction in Gr/Al_2_O_3_/Si solar cells is influenced not only by the Al_2_O_3_ film but also by the Al_2_O_3_/Si interface layer. These effects will be discussed in Section 4. Finally, concluding remarks will be provided in Section 5.

## 2. Photon Sensitivity of Graphene/Silicon Interface

**Figure 1:**
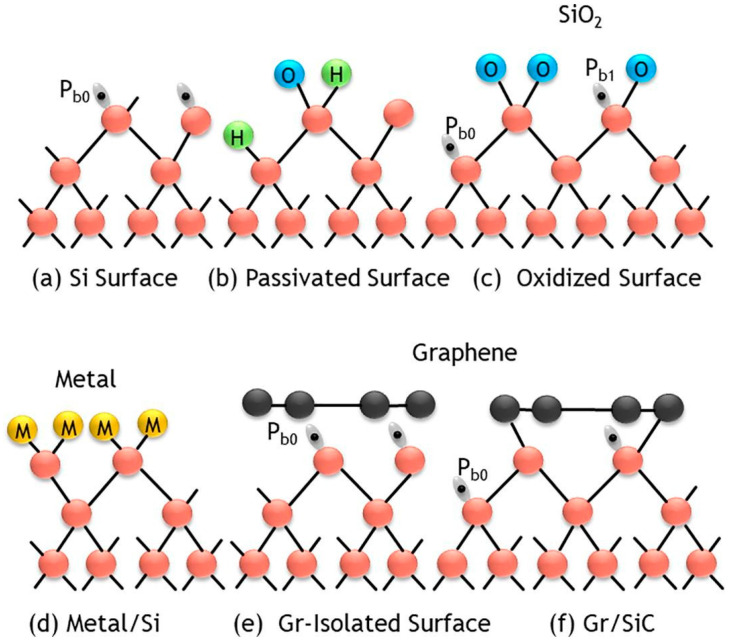
Illustration of Si surface interfacing to different materials: (**a**) native silicon surface; (**b**) silicon surface with hydrogen and hydroxyl passivation; (**c**) oxidized silicon surface; (**d**) metal-covered Si surface; (**e**) native silicon surface covered by graphene; (**f**) Si-carbon covalent bonding [4]. © 2021 Elsevier. Reproduced with permission.

It is rarely understood that the poor power efficiency of the Gr/Si solar cell mostly originated from the high amount of silicon dangling bonds on the silicon surface. Figure 1 compares the native silicon surface and its interfacing with different materials. Unlike the metal/Si and oxide/silicon interface, where the silicon dangling bond can effectively be passivated with the metal or forming silicon oxide, the graphene/Si contact is weakly bonded by van der Waal forces and the Si surface dangling bonds covered by graphene are electrically active and can trap both electrons and holes [4]. The Gr/Si Schottky diode was found to have high sensitivity to light illumination. Similarly to other pn junction-based photodetectors, the working principle of the Gr/Si Schottky diode is usually attributed to the carrier generation from the depletion layer in the silicon side. With this mechanism, photons are first absorbed in graphene and result in the generation of high-energy electrons. Under reverse bias, the high-energy electrons emit over the Schottky barrier and, in turn, cause impact ionization in the silicon depletion layer [21,22]. Hence, a reversely biased Gr/Si Schottky has a higher photocurrent than the forward ones because of the larger depletion layer width and the biasing. Several unexplained effects were reported. It was reported that in some cases, the photon-generated current shows a saturation trend similar to the temperature characteristics. The observation does not seem to agree with the impact ionization model as proposed. It was suggested that the surface silicon defects (P_b0_ centers) should involve the charge transport and affect the I–V characteristics of the Gr/Si Schottky diode. The P_b0_ centers can trap both electrons and holes, and the charge trapping-detrapping processes are strongly temperature-dependent [23]. This work reports on the light-dependence and hysteresis of forward I–V characteristics arising from the silicon surface defects.

**Figure 2:**
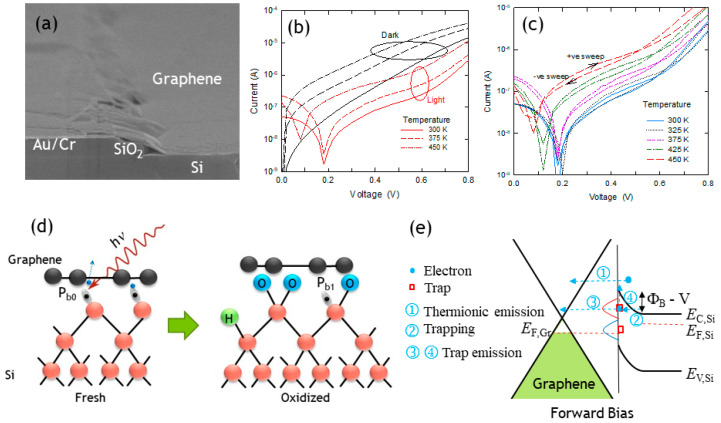
(**a**) SEM on the cross-sectional view of the graphene/Si structure; (**b**) Comparison of the forward current–voltage characteristics in dark and under light illumination. (**c**) Bi-directional sweep for the I–V measurement showing hysteresis effect. (**d**) Proposed photon-assisted silicon surface defect detrapping model for graphene/Si Schottky junction under light illumination (**left**) and the suppression of photon effect by surface oxidation (**right**). (**e**) Band diagram of graphene/Si structure showing the possible involvement of acceptor-like and donor-like silicon P_b0_ centers in the current conduction.

Figure 2a shows the SEM picture of a cross-sectional view of the device. Graphene/Si photodetectors are usually operated at reverse bias where the dominating current component is due to the impact ionization of the Si depletion region. To better reveal the effect involving the Si surface defects, we study the forward I–V characteristics where the photocurrent component should be much smaller. Figure 2b compares the forward I–V characteristics measured at dark and under room light (fluorescent tube) illumination. It is unexpected that the forward current measured at dark is much larger than under light illumination. One may expect that there should be higher carrier density under photon illumination; the forward current turns out to be smaller, however. This effect can be explained by the change in the effective Schottky barrier under light illumination, which gives rise to trapped charge depopulation. The surface of a three-dimensional crystal has a high amount of silicon dangling bonds, which are known as P_b0_ centers (See Figure 2d). The 2D graphene surface may still have some surface defects, but the density should be much smaller than the silicon one. The Gr/Si contact was maintained by the weak van der Waals force, the P_b0_ centers on Si surface defects are not chemically passivated and remain electrically active. Hence, the graphene/Si barrier height cannot be simply modeled with the Schottky–Mott equation. The P_b0_ centers can trap both electrons and holes [23]. The P_b0_ centers can be passivated with thermal oxidation (right figure of Figure 2d). Because of the existence of the air gap and air bump, significantly different characteristics were found on freshly prepared and long-term stored samples [4]. These phenomena and the current light dependent characteristics can be attributed to the effect of P_b0_ centers. At dark and under forward bias, a large amount of the surface traps (accepted like traps) are filled with electrons and that increases the surface Fermi level. As a result, the Schottky barrier is smaller and leads to a larger forward current at dark. Under light illumination, the trapped electron can be excited by the photons (See Figure 2d). The Fermi level is smaller and, thus, a larger potential barrier at the Gr/Si interface. Although the bulk carrier concentration of the Si substrate may be higher under photon illumination, the forward current is still smaller. We further conducted a bi-directional scan for the measurement of the forward characteristics (Figure 2c). Hysteresis was observed for all temperatures. As compared with the reverse sweep (decreasing sweep voltage in measurement), the forward sweep results in a higher current for bias voltage smaller than about 0.6 V. A reverse relationship was found for V > ~0.6 V. The hysteresis became larger as the temperature increased because of the higher thermionic emission rate. Again, the effect can be consistently explained with the charge trapping–detrapping involving the surface P_b0_ centers. In addition to the normal Schottky current via mechanism 1 in Figure 2e, the forward sweep results in electron trapping via mechanism 2, and that lowers the Schottky barrier. A larger forward voltage can result in the charge detrapping (mechanism 3 in Figure 2e) and give rise to a larger barrier during the reverse sweep. The charge detrapping and thermionic emission can be enhanced at higher temperatures and that explains the temperature effects.

## 3. Graphene/Silicon Interface Passivation Layer

Considering the nature of the Gr/Si interface discussed in Section 2, it can be affirmed that growing a high-quality insulating layer is the most effective way to passivate silicon dangling bonds and significantly enhance the power conversion efficiency (PCE) of solar cells. Various materials were considered, with Al_2_O_3_ emerging as one of the best candidates. The characteristics improvement was misattributed to the oxygen vacancy reduction in the recent report. The tools applied may not have been the most suitable, and the interpretation could be considered inaccurate. In addition to effectively chemically passivating the silicon surface state density, the negative fixed oxide charge associated with the Al_2_O_3_ film introduces an additional field effect near the silicon surface, which was not recognized in previous works. Furthermore, the impact of interface roughness on the ultrathin insulator layer could be significant. These issues were not sufficiently addressed.

By conducting an XPS survey on Al_2_O_3_, Kim et al. identified O 1s peaks at energies of 531 eV and 532.5 eV, which were assigned to lattice oxygen and oxygen vacancies, respectively [16]. However, the correlation of the O 1s signal to oxygen vacancies is doubtful, making the proposed mechanisms unfounded. XPS is not a suitable method for detecting oxygen vacancies. Moreover, ALD oxide films should have very low levels of oxygen vacancies, and it is unlikely for oxygen vacancies to exceed 9% and reach up to 19.8% as reported, unless the film is deposited under highly reducing conditions. Instead, the 532.5 eV peak is usually attributed to SiO_x_ and silicate bonds. We reproduced the Gr/Al_2_O_3_/Si structure and conducted a further in-depth analysis of both the XPS and current–voltage characteristics. A 3 nm thick Al_2_O_3_ film was grown on an HF-last silicon substrate using trimethylaluminum (TMA) and water vapor as precursors. The chamber pressure was 1.3 mbar, and the substrate temperature was 200 °C. Post-ALD thermal annealing at 300 °C for 1 min was conducted. It should be noted that the ALD of Al_2_O_3_ on silicon usually starts with hydrogen-terminated Si substrates. Al_2_O_3_ phases have difficulty achieving reliable nucleation on an HF-last Si surface. The H_2_O cycle should start first, resulting in the formation of an interfacial transition layer, which could negatively impact the ultrathin Al_2_O_3_ film. In the rest of this section, we will elaborate on this issue in detail.

**Figure 3:**
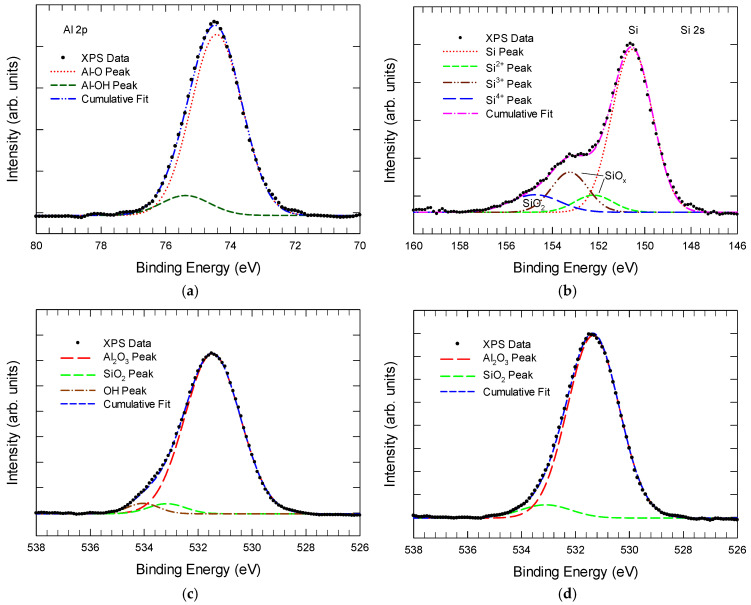
(**a**) Decomposed Al 2p spectrum of the Al_2_O_3_ film showing the Al-OH component. (**b**) Si 2s XPS spectrum taken at the Al_2_O_3_/Si interface, showing the components of SiO_2_ and SiOx phases. (**c**) Three oxygen bonding states were found in the as-deposited Al_2_O_3_ sample. (**d**) Post-metallization annealing (PMA) at 300 °C resulted in a significant reduction in the OH peak.

Figure 3a shows the Al 2p spectrum. The main peak is centered at 74.4 eV, corresponding to the binding energy of the Al-O bond. Gaussian decomposition reveals a higher energy sub-peak at 75.4 eV, which can be attributed to Al-OH bonding. The presence of Al-OH groups could result in an excess amount of oxygen in the film. No lower binding energy corresponding to Al-Al bonds was observed, indicating that the amount of oxygen vacancies in the ALD film is likely low. Figure 3b presents the Si 2s spectrum taken with a takeoff angle of 20°, representing the Si signal from the Al_2_O_3_/Si interface. The Si 2s spectrum can be decomposed into three peaks: the Si-Si peak at around 150.5 eV, and two suboxide peaks at 152.1 eV and 153.2 eV. The peak at 154.6 eV is attributed to the SiO_2_ phase. Although the ALD Al_2_O_3_/Si interface is often considered almost completely abrupt compared to other deposition techniques, an interface transition region still exists. This interface layer could arise from the native oxide of the silicon substrate. Even with the HF-last process used here, the surface will undoubtedly contain many dangling bonds, which provide favorable conditions for -OH group adhesion during the early cycles of ALD growth.

Figure 3c shows the interfacial O 1s XPS spectrum for the as-deposited sample with Gaussian decomposition. The spectrum can be decomposed into three peaks at energies of 531.4 eV, 532.9 eV, and 534 eV. We attributed the 531.4 eV peak to the oxygen-binding energy in Al-O, which is widely accepted. The 532.9 eV and 534 eV peaks are attributed to the SiO_2_ phase and OH groups (including Al-OH), respectively [18]. After thermal annealing at 300 °C for 1 min (see Figure 3d), although there are no notable changes in film thickness, the 534 eV peak is reduced, and the 532.9 eV peak is enhanced. This indicates interface oxidation and the release of hydrogen during annealing. Both the Si 2s and O 1s peaks indicated that the amount of interfacial SiO_x_ slightly increased after 300 °C annealing. Additionally, the Al 2p and O 1s spectra together suggest the presence of OH groups in the film. Thus, the ALD growth of Al_2_O_3_ on HF-last silicon unavoidably introduces interface OH groups, resulting in Al-OH and Si-OH bonds. With thermal annealing, Si-OH transforms into SiO_2_, leading to a rougher interface.

It is worth noting that some researchers may have inadvertently identified the O 1s spectrum in the energy range of 532 to 534 eV as oxygen vacancies, which could lead to potentially questionable conclusions. [24,25,26,27]. There is no direct correlation between the oxygen vacancy with O 1s XPS signal [25,26,27]. In the work by Kim et al. [16], they assigned the main O 1s component at around 531 eV (O lattice) to Al-O bonding. The minor component in the energy range of 532.5 to 534 eV was attributed to oxygen vacancies or non-lattice oxygen. They further reported that the intensity ratios of lattice oxygen to “defect oxygen” were 9.0, 14.3, 12.7, and 19.8, respectively, for simple Al_2_O_3_ layers, NH_3_-grown Al_2_O_3_, H_2_O_2_-grown Al_2_O_3_, and NH_3_-H_2_O_2_ dual precursor Al_2_O_3_ layers. They concluded that using either NH_3_ or H_2_O_2_, especially when both precursors are used simultaneously, could reduce the amount of defective oxygen and non-lattice oxygen in Al_2_O_3_. They also suggested that “defects associated with oxygen vacancies and non-lattice oxygen can affect the charge transfer resistance and electron recombination in the Al_2_O_3_ interfacial layers, which can also influence the efficiency of the solar cell”. However, no further explanation was provided on why different precursors lead to significantly different levels of oxygen vacancies.

The process-dependent characteristics observed in ALD Al_2_O_3_ with different precursors can be explained as follows. Unlike other deposition techniques, in the HF-last silicon process, the H_2_O/H_2_O_2_ pulse must be applied in the first ALD cycles, resulting in the incorporation of OH groups on the freshly cleaned H-passivated silicon surface. This leads to the formation of Si-O bonds or a suboxide layer, which can grow further during subsequent high-temperature processing steps. Additionally, ALD growth on silicon typically starts imperfectly in an island growth mode via the absorbed OH groups. Thus, the first few AlO_x_ monolayers are imperfect. Once the AlO_x_ layer reaches a certain thickness, e.g., 0.5 to 1 nm, stoichiometric growth of Al_2_O_3_ occurs. During thermal annealing or later high-temperature steps, such as the 550 °C for 2.5 h during the PECVD growth of the graphene layer as prepared by Kim et al. [16], the suboxide layer or silicate layer may grow thicker. For the ALD group with NH_3_ and H_2_O_2_ dual precursors, NH_4_OH is formed, which is mildly corrosive to SiO_x_ and Al_2_O_3_. This causes the initial SiO_x_ and AlO_x_ phases to be etched away. Additionally, it makes the surface OH-terminated, rendering it more suitable for ALD growth. This process results in a sharper and more uniform Al_2_O_3_/Si interface, leading to a smaller 532.5 eV O 1s peak. Based on our proposal, the high “Lattice O/Defect O” XPS peak ratio of the NH_3_-H_2_O_2_ sample implies a low interface SiOx or silicate content, indicating that the NH_3_-H_2_O_2_ precursors can improve the Al_2_O_3_/Si interface for Al_2_O_3_ deposition. This has nothing to do with oxygen vacancies. The TEM image provided in the paper by Kim et al. shows a sharp Al_2_O_3_/Si interface, which is better than in many other reports [16]. However, the etching effect persists in the subsequent NH_3_ and H_2_O_2_ pulses for Al_2_O_3_ deposition. Consequently, more Al vacancies, which are triple-negatively charged, will be produced with the NH_3_-H_2_O_2_ dual precursors. Al_2_O_3_ was found to have a higher fixed negative charge density, ranging from over 10^12^ to 10^14^/cm^2^. The capacitance-voltage (C-V) measurement can be used to quantify the fixed oxide charge density in an insulator.

## 4. Current Conduction Mechanisms

In many recent reports on Gr/Insulator/Si solar cells, the current–voltage characteristics were fitted, without any modification, with the Schottky equation with ideality factor, *n*, which is

(1) $$J=A^{*}T^{2}exp\left(-\frac{\Phi_{SB}}{kT}\right)exp\left(\frac{qV}{nkT}\right)$$

In these studies, the ideality factor and Schottky barrier height, Φ*_SB_*, are often taken as device performance indicators which were extracted from the measured I–V characteristics as [13,16]:

*n* = (*q/k_B_T*) (d*V*/dln(*J*)) (2)

Φ*_SBH_* = ln(A^*^T^2^/*J_rev_*)*k_B_T* (3)

where *k_B_* is the Boltzmann constant, *q* is the elementary charge, *J_Rev_* is the reverse saturation current density, A* is the Richardson constant, which is 252 A·cm^−2^ K^−2^ for Gr/n-type Si barrier, and *T* is the absolute temperature.

The validity and technical implications of these parameter extractions are questionable. The primary reason is that these devices are not Schottky diodes but MIS capacitors. As ensuring the silicon surface is oxide-free during the non-in situ graphene layer deposition process is challenging, the van der Waals gap should be considered a tunneling layer rather than a hybridized contact even if direct contact of graphene on silicon was taken [28]. Treating MIS capacitors as Schottky diodes originated from a work by Card and Rhoderick [29]. They demonstrated that when the insulator layer is thin enough to allow direct tunneling of carriers, the current–voltage characteristics of the MIS structure can be approximated by the Schottky barrier equation. This approximation involves modifying the Richardson coefficient with a transmission coefficient that depends on the barrier height between the silicon and the insulator and the insulator thickness. The modified Richardson coefficient is [29]:

(4) $$A^{*}=A_{0}\exp(-\sqrt{\chi }\delta )$$

where *A*_0_ is the original Richardson constant, χ, the mean barrier height value between the insulator and the semiconductor, and δ is the tunneling oxide thickness which must be thinn enough so that direct tunneling can occur.

**Figure 4:**
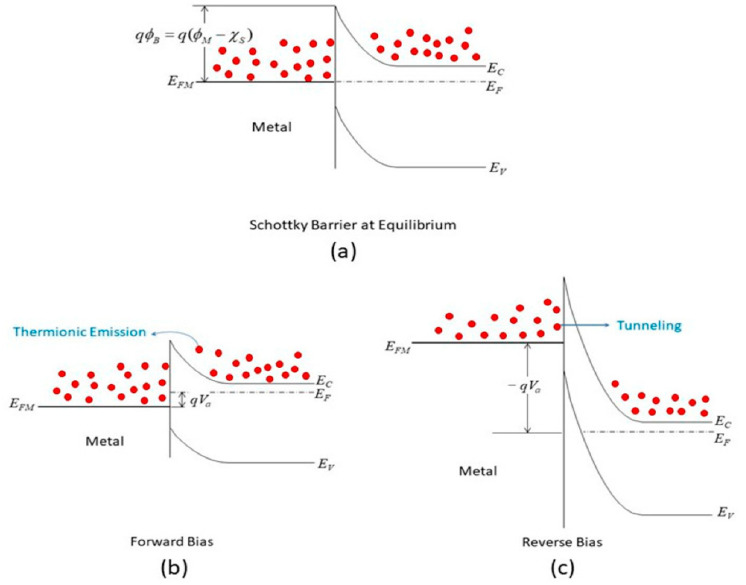
Band diagram and conduction mechanism of Schottky contact under (**a**) equilibrium, (**b**) forward bias; and (**c**) reverse bias. Reproduced from [28].

Unfortunately, this important modification in (4) and the assumptions being used were often neglected in the literature [28,30]. Yet the current–voltage characteristics of graphene/Si diode are still able to be described properly with the elementary Schottky equation considering the gap of the van der Waal contact is so thin that direct tunneling over the gate is a suitable approximation. However, the Schottky conduction model might not be the most suitable approach for the MIS structure of surface-passivated MIS solar cells. In addition, one should also note that the approximation is only applicable for forward current as the barriers at the metal/insulator and silicon/insulator interfaces are different. Thus, using (3) with the Richardson coefficient of 252 A·cm^−2^ K^−2^ (for the Gr/Si interface) and the reverse saturation current (*J_rev_*) to calculate the barrier height of the MIS structure would lead to inaccurate results [30]. The coefficient should be adjusted with the Si/insulator barrier height and insulator thickness. In addition, it is important to note that, even when using the Schottky model, the reverse current is better modeled by direct tunneling rather than the Schottky equation under reverse bias. Figure 4 illustrates the band diagram and the current conduction mechanisms for a metal/n-type semiconductor Schottky contact under both forward and reverse bias conditions.

**Figure 5:**
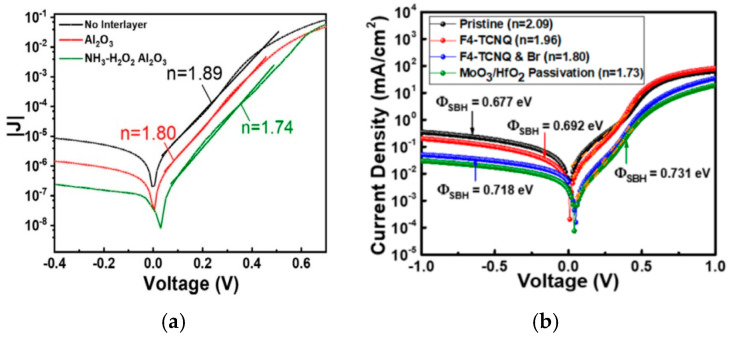
(**a**) Illustration of the “Ideality Factor” evaluation of three different graphene/Al_2_O_3_/Si MIS Schottky diodes reported by Kim et al. [16]. Large gaps for the linear fitting in the forward bias region were noted. © 2022 Elsvier. Reproduced with permission. (**b**) Ideality factor and Schottky barrier calculated by Kadam et al. [13]. © 2023 American Chemical Society. Reproduced with permission.

Figure 5 presents examples of inappropriate modeling, fitting, and characterization of MIS solar cells with different graphene/Si interlayers. As shown in Figure 3a, Kim et al. observed that the Schottky barrier, extracted using Equation (3), changes from 0.732 eV to 0.816 eV, and the ideality factor varies from 1.74 to 1.89 for an approximately 1nm thick Al_2_O_3_ interlayer [16]. Kim et al. attributed the larger *n* value of 1.89 to recombination loss due to parasitic resistance and thermionic emission, and the smaller *n* values to the reduction in interface trap states [16]. However, the fitting error is significant, and the fittings have a certain degree of arbitrariness. The curves are plotted on a logarithmic scale, so a slight difference means a significant error. Moreover, the *n* value can vary by 10 to 20 percent depending on the date range chosen, because the curves are not linear in general. The author also claimed that the *y*-axis intercept of the extrapolated line in the ln(*J*)-*V* plots is the saturation current density, which is 8.74 × 10^−6^ mA/cm^2^, 1.42 × 10^−6^ mA/cm^2^, and 2.41 × 10^−6^ mA/cm^2^, for the three samples, respectively. This was the way to find the zero bias current of the Schottky diode [16]. However, it is obvious that these fitted values are about one magnitude higher than the measured currents at zero bias. This indicates that the current–voltage characteristics do not follow the Schottky equation.

In an MIS solar cell with a thinner insulator (~2 nm HfO_2_), produced by Kamdam et al. [13], the ideality factor ranges from 1.73 to 2.09, and the barrier height changes from 0.677 eV to 0.713 eV (see Figure 5b). We note that the forward region exhibits a higher degree of non-linearity, with the forward current appearing to be composed of two exponentially increasing currents rather than a simple Schottky current. The current–voltage characteristics are better described by tunneling currents [30]. In these studies, the “ideality factor” was used as a performance indicator, even though the I–V characteristics did not fit well with the Schottky equation.

The ideality factor, *n*, was initially introduced in pn junction diodes. It has a specific physical interpretation: when the forward current is solely due to diffusion, *n* = 1; if carrier generation and recombination (GR) processes are involved, *n* = 2; and *n* can rise to 4 if multiple defect energy levels participate in the GR process. Therefore, the case of *n* = 1 signifies an ideal diode, while *n* > 1 indicates the presence of GR current [28]. In this context, the ideality factor accurately reflects the quality of the pn junction diode. However, in MIS diodes, although the “ideality factor” might enhance voltage dependence fitting in certain cases, it does not serve as an indicator of junction quality and lacks sound physical or technical implications in these scenarios. For both graphs shown in Figure 5, it is noted that the reverse region exhibits an abnormally high current compared to a real Schottky diode. Furthermore, a linear relationship between ln(*J*) and *V* was found at larger reverse voltages, suggesting that direct tunneling is the dominant mechanism for the reverse current [28,30]. Thus, one can infer that the forward current should also be governed by tunneling, but with different barrier heights.

**Figure 6:**
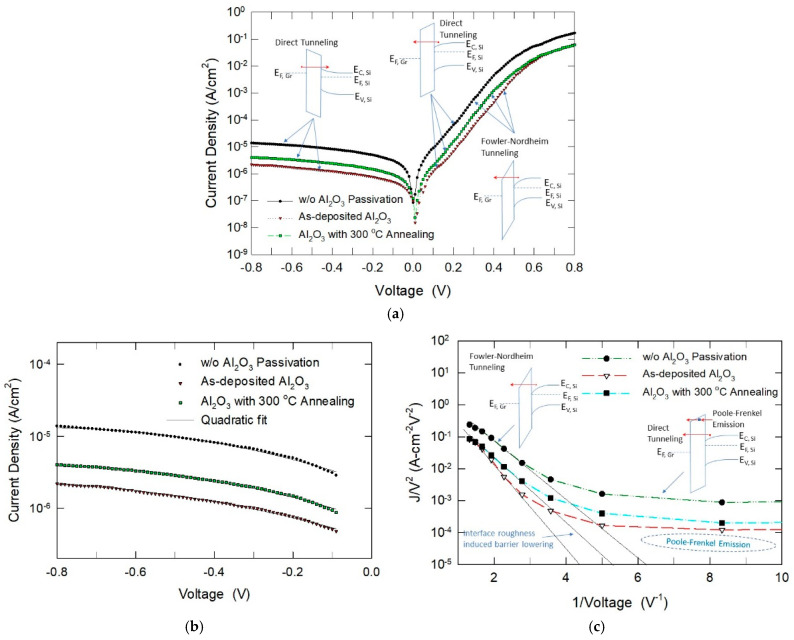
(**a**) Measured current–voltage characteristics of graphene/Al_2_O_3_/Si MIS diodes and proposed conduction mechanisms for three different biasing conditions. (**b**) Reverse characteristics fit well with the quadratic equation, indicating the conduction is due to direct tunneling. (**c**) Fowler–Nordheim plot of the forward characteristics suggests that the current conduction is due to FN tunneling at high voltage.

Here, we conduct a similar experiment on Gr/Al_2_O_3_/Si solar cells. The results are depicted in Figure 6. Figure 6 displays the current–voltage characteristics of our samples, which is more similar to those shown in Figure 5a. The Al_2_O_3_ film was ALD growth on HF-last silicon and the thickness was about 3 nm also. As discussed earlier, it seems the I–V relationship does not follow the Schottky thermionic emission model. The reverse behavior exhibits a tunneling current characteristic rather than that of a Schottky diode under reverse bias. At high reverse bias, the reverse currents rise exponentially with the applied voltage. As previously noted, the Schottky diode model by Card and Rhoderick [29] is not applicable to the MIS diode in reverse bias, which is dominated by direct tunneling. The direct-tunneling current can be analytically described using the Wentzel-Kramers-Brillouin (WKB) approximation for barrier transparency [31,32]. In the large reverse bias region, ln*J_DT_* ∞ *V*, namely, the slope of the ln*J_DT_* versus voltage plot is a linear function of the applied voltage [32]. That is, the tunneling current at low voltage can be fitted well with a quadratic equation (see Figure 6b).

Considering the reverse current is due to direct tunneling and the forward current at high voltage is due to FN tunneling, it is natural to assume the forward current before the onset of the FN tunneling is due to direct tunneling. Figure 6c plots the forward characteristics in the Fowler–Nordheim relationship, which is quoted in (5) [33].

(5) JFN=AE2exp⁡(−BE)

where *E* is the applied electric field, and *A* is a proportional constant. *B* in (5) is the FN slope, which is

(6) $$B=\frac{4}{3}\frac{{{(2m}_{i})}^{1/2}}{q\hbar }{\Phi_{B}}^{3/2}$$

where *m_i_* is the electron mass in the oxide, ℏ the reduced Planck’s constant, and Φ*_B_* the barrier height between the silicon and the oxide.

**Figure 7:**
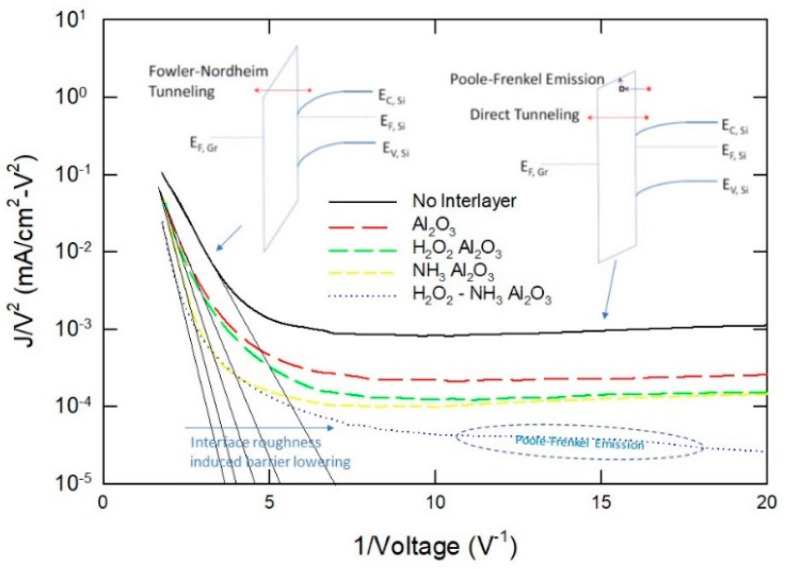
Fowler–Nordheim plot of the forward current–voltage characteristics at large forward bias for MIS structures with Al_2_O_3_ prepared by different precursors. Data taken from Ref. [16].

At high voltage (left-hand side of the *x*-axis), a linear region was observed, indicating the current conduction in this region should be due to the FN conduction. An inset further explains this mode of conduction. At a large biasing voltage, a severe band bending results in more electrons tunneling through the triangular region of the barrier (see Figure 6c). According to (6), the FN slope depends only on the barrier height when the effective mass and film thickness are constant. At low bias voltage (right side of the *x*-axis), the current conduction occurs by direct tunneling over the interlayer (see band diagram depicted in Figure 6a). Similar phenomena were revealed with the experimental current–voltage characteristics presented by Kadam et al. [13]. The data from Kim et al. [16] also follow the FN and PF models. As shown in Figure 7, we can infer that the barrier height for graphene/silicon direct contact is the lowest, while the H_2_O_2_-NH_3_ Al_2_O_3_ sample has the highest barrier height. The Al_2_O_3_ interlayer prepared by four different precursors shows different FN slopes in the ln(*J*/*V*^2^) versus *V*^−1^ plot, which may be partly due to the different film thickness. However, if one assumes that all the samples have the same thickness, then the different barrier heights should be mainly caused by the different interface roughness [34,35,36,37]. As discussed earlier, a sample prepared by H_2_O_2_ precursor has a high content of SiO*_x_* and AlO*_x_* suboxides, which make the Al_2_O_3_/Si interface rougher and lower the interface barrier, resulting in a larger leakage current [34,35,36,37]. Whereas the sample prepared by the H_2_O_2_-NH_3_ combined precursor can remove the SiO*_x_* and AlO*_x_* in the first few cycles. The Al_2_O_3_/Si interface is smoother and has less roughness-induced barrier lowering; therefore, the H_2_O_2_-NH_3_ sample has a higher barrier and a smaller leakage current.

We noted that the current conduction of the H_2_O_2_-NH_3_ sample deviated slightly from the direct tunneling characteristics at a small forward bias. We tentatively attributed this behavior to the Poole–Frenkel emission via the Al vacancies [20]. However, the Poole–Frenkel currents in these samples were negligible when compared to the direct tunneling current. The Poole–Frenkel current only became notable when the tunneling barrier was high. This suggested that the films prepared by Kim et al. should have a low defect density. Among all the samples, the H_2_O_2_-NH_3_ sample showed the largest positive shift in the I–V curve, indicating that it had the highest amount of Al vacancies. Therefore, we are inclined to believe that the negative oxide charge, rather than the reduction in oxygen vacancies suggested by Kim et al. [16], is responsible for surface recombination suppression and power conversion efficiency (PCE) enhancement. The negative charges in Al_2_O_3_ prevent the electrons from recombining with the silicon surface dangling bonds and facilitate the hole injection from silicon [20]. This important fact was ignored in the report by Kim and co-workers [16].

In short, results from various sources and processes consistently indicate that current conduction in an MIS solar cell is better described by direct tunneling and Fowler–Nordheim emission. This suggests that the Schottky barrier, which measures the difference in Fermi level and electron affinity between graphene and the silicon substrate, cannot be accurately determined from current–voltage characteristics. Consequently, the previously reported values [13,16] lack strong technical implications. Additionally, since the current is due to tunneling, the ideality factor, which typically indicates the contribution of diffusion and recombination currents, is not an appropriate figure of merit in this context.

## 5. Conclusions

In summary, we have identified several issues in previous studies and provided better interpretations with stronger physical grounding regarding the characteristics of graphene-based MIS solar cells. The efficiency of graphene/silicon Schottky junction solar cells was limited by the electrically active silicon surface dangling bonds. Significant performance improvement was achieved through proper surface passivation with a thin tunneling oxide. However, this passivation layer causes the current conduction to deviate from the conventional Schottky equation. Therefore, the Schottky barrier and ideality factor are no longer valid performance indicators for MIS structure solar cells.

In the MIS structure, the current conduction is governed by direct tunneling in reverse bias, Poole–Frenkel emission at small forward bias, and Fowler–Nordheim conduction at a large forward bias. These characteristics are process- and material-dependent. We disagree with previous work suggesting that oxygen vacancies play a significant role in improving the energy efficiency of Graphene/Al_2_O_3_/Si solar cells. Instead, we propose that the negative fixed charge induced by triple-charged aluminum vacancies, which can reduce surface non-radiative recombination, should play an important role. In addition, the silicon surface and ALD precursor could lead to different interface roughness, due to the formation of SiO_x_ phases, and affect the value of the tunneling barrier height. These issues are crucial for better understanding the device physics and optimizing the process to enhance the long-term stability and power-conversion efficiency of graphene–silicon-based solar cells.

## Data Availability

The original contributions presented in the study are included in the article, further inquiries can be directed to the corresponding author.
